# Editorial: Diabetic kidney disease: routes to drug development, pharmacology and underlying molecular mechanisms, volume II

**DOI:** 10.3389/fphar.2025.1609100

**Published:** 2025-04-25

**Authors:** Divya Bhatia, Swayam Prakash Srivastava

**Affiliations:** ^1^ Division of Nephrology and Hypertension, Joan and Sanford I. Weill Department of Medicine, Weill Cornell Medicine, New York, NY, United States; ^2^ Life Sciences Institute, University of Michigan, Ann Arbor, MI, United States

**Keywords:** diabetic kidney disease, sirtuin 3, glucocorticoid receptor, fibroblasts growth factor receptor 1, angiopoeitin like protein 4, kidney failure, endothelial-to-mesenchymal transition, kidney fibrosis

## Introduction

Diabetic kidney disease (DKD) is the leading cause of chronic kidney disease (CKD), driven primarily by metabolic dysfunction, immune cell infiltration, and inflammation in the kidney. These changes lead to nephron loss, mesenchymal activation, and finally kidney fibrosis (Li and Susztak, 2025). DKD accounts for almost half of all CKD cases, with around 40% of individuals with diabetes developing DKD (Li and Susztak, 2025; Cooper and Warren, 2019). Despite its prevalence, DKD progression varies among patients, and its underlying mechanism remains poorly understood. DKD often remains asymptomatic until it advances to kidney failure. Kidney fibrosis is the final pathological outcome of CKD in the diabetic kidney. Several theories have been proposed regarding the origin of myofibroblasts, which play crucial roles in the progression of kidney fibrosis. Myofibroblasts can derive from tubular epithelial cells via partial epithelial-to-mesenchymal transition (EMT), endothelial cells through endothelial-to-mesenchymal transition (EndMT), or profibrotic (M2-like)-macrophages via macrophage-to-mesenchymal transition (MMT) (Lovisa et al., 2020; Bhatia et al., 2019). Additionally, myofibroblasts have also been proposed to originate from activated resident fibroblasts or bone marrow-derived cells (Srivastava et al., 2019; Srivastava et al., 2020a). In DKD, an increase in endothelial cell permeability, and a decrease in pericytes coverage contribute to tissue damage and the release of chemokines further exaggerate the infiltration of immune cells and inflammation. The excessive inflammation-mediated tissue injury results in the deposition of the components of extracellular matrix (ECM), such as collagens, fibronectin, desmin, and integrins. Ultimately, these pathological alterations disrupt kidney structure, function, and metabolism, resulting in kidney fibrosis and failure (Rayego-Mateos et al., 2023). A recent study highlighted a comprehensive multimodal atlas of the human kidney (Li et al., 2024). Using single-cell assay for transposase-accessible chromatin and RNA sequencing (scATAC-seq) and spatially resolved metabolomics, this study has characterized region-specific metabolic signatures of different nephron segments, including endothelial cells and proximal tubular epithelial cells (Li et al., 2024). Spatial metabolomics further demonstrated suppressed fatty acid oxidation (FAO) along with an accumulation of long-chain acylcarnitines, a type of lipids that are key intermediates in FAO, in proximal tubular cells from patients with kidney disease (Kang et al., 2015; Mukhi et al., 2023). Related research in type I diabetic (T1DM) patients identified that despite preserved kidney function and the absence of albuminuria at an early stage, metabolic disturbances can be present (Choi et al., 2024). Utilizing single-cell RNA sequencing, spatial metabolomics, and positron emission tomography imaging to assess tricarboxylic acid cycle (TCA) cycle activity, they reported suppressed oxidative metabolism in the renal cortex and medulla of T1DM patients (Choi et al., 2024). Overall, this study highlights that early metabolic impairments could serve as preclinical markers for DKD, suggesting a critical role of mitochondrial dysfunction in the progression of DKD. Elevated urinary lactate levels have emerged as a biomarker of mitochondrial dysfunction and correlate with a decline in kidney function in diabetic patients (Darshi et al., 2024). Mitophagy, the mitochondrial quality control program that recycles defective mitochondria is also impaired during DKD (Bhatia et al., 2019; Bhatia et al., 2020; Bhatia and Srivastava, 2023). Mitochondrial proteome data profiling in healthy podocytes revealed that NADH:ubiquinone oxidoreductase subunit S4 (NDUFS4) stabilizes respiratory supercomplexes. In diabetic podocytes, suppressed NDUFS4 levels were also associated with reduced mitochondrial function, contributing to DKD progression (Mise et al., 2023).

Here, we discussed two sections:

## New cellular and molecular mechanisms

**FIGURE 1:**
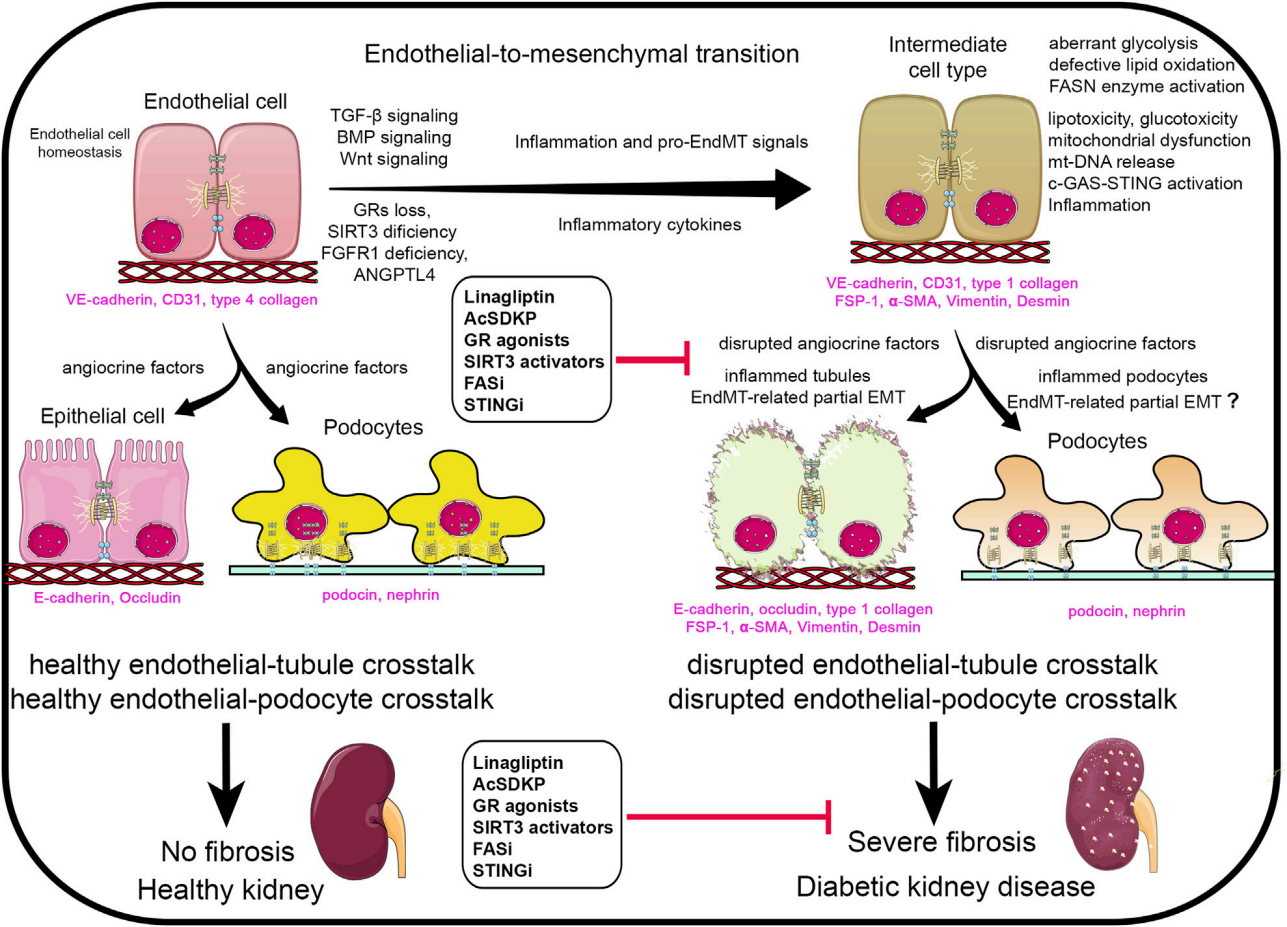
Renal angiocrine factors regulating pro-endothelial-to-mesenchymal transition (EndMT), pro-epithelial-to-mesenchymal transition (EMT) and pro-inflammatory signals. Abbreviations- TGF-β: Transforming growth factor-β.; GRs: glucocorticoid receptors.; BMP: Bone morphogenetic protein.; FGFR1: fibroblasts growth factor receptor 1.; ANGPLT4: angiopoietin like-4 protein.; AcSDKP: N-seryl-acetyl-lysyl proline.; FASi: fatty acid synthetase inhibitor.; STINGi: STING inhibitor.; mt-DNA: mitochondrial DNA.; FSP-1: fibroblasts specific protein 1.; α-SMA: α-smooth muscle actin. Components of this figure were created using Servier Medical Art templates, which are licensed under a Creative Commons Attribution 3.0 Unported License; https://smart.servier.com.

Suppressed FAO in diabetic renal tubules, endothelial cells, and podocytes is concomitantly associated with aberrant glycolysis and elevated lactate levels (Srivastava et al., 2018). This metabolic reprogramming is believed to generate metabolites that sustain myofibroblasts’ survival, contributing to fibrosis progression (Srivastava et al., 2018). This mesenchymal-metabolic shift, characterized by a gain of mesenchymal activity due to altered metabolism, is likely driven by mitochondrial dysfunction (Srivastava et al., 2021a). Targeting mesenchymal metabolic shift represents a potential therapeutic strategy for DKD (Srivastava et al., 2021a). In recent years, our group has demonstrated the anti-EndMT, anti-inflammatory, and anti-fibrotic effects of endothelial SIRT3, endothelial glucocorticoid receptors, and endothelial fibroblasts growth factor receptor 1 in diabetes (Srivastava and Goodwin, 2023; Srivastava et al., 2021b; Srivastava et al., 2021c; Li et al., 2020a). These anti-fibrotic effects are accompanied by improved mitochondrial function and structure, reduced inflammation, attenuation of mesenchymal metabolic shifts and partial EMT in the kidney tubules. Moreover, we reported that mitochondrial fusion, biogenesis, mitophagy, and antioxidant defense in the kidney are suppressed while fission is induced during kidney fibrosis (Bhatia et al., 2019; Bhatia et al., 2022). Mitochondrial quality control program helps in attenuating kidney macrophage-derived inflammation and progression of kidney fibrosis (Bhatia et al., 2019; Bhatia et al., 2022). Apart from the suppressed fatty acid oxidation, *de novo* lipogenesis (DNL) plays a critical role in kidney fibrogenesis (Mukhi et al., 2023; Srivastava et al., 2024). In diabetic tubules and podocytes, key DNL enzymes, including fatty acid synthase (FASN), acetyl-CoA carboxylase (ACC), perilipin and sterol regulatory element-binding protein 1 (SREBF1) are upregulated. The inhibition of FASN using a small molecule inhibitor FASNALL suppresses DNL and mitigates renal fibrogenesis in diabetic mice (Mukhi et al., 2023; Srivastava et al., 2024). Angiopoietin like-4 (ANGPTL4), a secretory protein, regulates lipid metabolism, FAO, and DNL by inhibiting lipoprotein lipase (LPL) in the kidney (Srivastava et al., 2024). While ANGPTL4 is chiefly secreted by adipose tissue and liver and mostly in a sialylated form (Clement et al., 2014; Clement et al., 2011). Kidney podocytes and tubules also secrete a hyposialylated form of ANGPTL4, which plays pro-proteinuric and fibrogenic roles in the diabetic kidney (Clement et al., 2011). The fibrogenic form of the ANGPTL4 functions independently of LPL (Srivastava et al., 2024). Excessive lipid flux suppressed FAO and increased DNL that contribute to lipid accumulation, and finally mitochondrial dysfunction in the tubular epithelial cells. Mitochondrial injury leads to the release of mitochondrial DNA (mtDNA) into the cytosol, where it activates the c-GAS-STING pathway and triggers tubulointerstitial inflammation (Bhatia et al., 2020). The inflamed tubules undergo mesenchymal activation, ultimately leading to tubulointerstitial fibrosis during DKD (Srivastava et al., 2024; Chung et al., 2019). Figure 1 demonstrate that healthy endothelial cells secrete healthy angiocrine factors that nourishes the other neighbouring cells and are important for the podocyte and tubule cell health. However, during inflammation which is associated to induce the pro-EndMT signals disrupts the endothelial cell metabolism in diabetes. Disruption of the endothelial cell metabolism leads to alterations in the angiocrine factors or secretion of unhealthy angiocrine factors which not only worsen the cells itself but the cell organelles, such as mitochondria. Therefore EndMT-derived angiocrine factors, disrupt the tubules cells and podocytes possibly through inflammation and partial EMT processes. However, the EMT in the podocyte is still needs further investigation for the researchers. Accumulative effects disrupt the essential crosstalk among endothelial cell, tubular and podocyte cell, finally leading to proteinuria, and severe fibrosis which is phenomenal feature of diabetic kidney disease.

In this Research Topic, a manuscript by Zhang et al. demonstrated that fibroblast growth factor (FGF)-21 by reducing glomerular damage, inflammation, and thrombosis while regulating the cell cycle through CDK1, exerts protective functions in diabetic nephropathy (DN). Additionally, Wang et al. described the role of SUMOylation in DKD and highlighted that natural product-mediated modulation of SUMOylation represents a potential therapeutic strategy for the treatment of DN. Yang et al. reported that N-myc downstream regulated gene 1 (*NDRG1*) modulates the progression of kidney fibrosis in DKD. Finally, Liu et al. studied a relationship between *SLC5A2* gene expression and estimated glomerular filtration rate (eGFR), wherein inhibition of *SLC5A2* was associated with higher eGFR.

## New therapeutics

A significant knowledge gap exists between basic biology and drug development, which contributes to the suboptimal treatment options for DN. A deeper understanding of DKD is urgently needed to develop novel therapeutics that can effectively suppress DKD progression at early stages. The currently available therapeutic regimens can retard, but not stop the progression towards kidney failure, and are often associated with side effects and intolerance. Thus, there is a pressing need for new therapeutic strategies to improve kidney function in diabetic patients. Additionally, standard clinical practices for managing DKD progression often fail to accurately prevent kidney function decline. Despite the critical importance of early detection and management of DKD, reliable diagnostic biomarkers and effective therapeutic targets remain lacking, presenting a major challenge for both clinicians and researchers. Therefore, there is an unmet need to explore new strategies for the diagnosis and management of patients with CKD. Though several therapeutic agents have been evaluated for their efficacy in preclinical models, preventing kidney fibrosis progression remains a significant challenge. Sodium-glucose cotransporter-2 (SGLT-2) inhibitor empagliflozin has been shown to attenuate kidney fibrosis via mitigating EMT and aberrant glycolysis, enhancing lipid oxidation and improving mitochondrial function by inducing SIRT3 in the mouse model of DKD (Li et al., 2020b). Another class of drugs, DPP-4 inhibitors such as linagliptin, has been reported to reduce kidney fibrosis via targeting profibrotic DPP-4-β1 integrin-mediated pathway in tubules and endothelial cells during DKD (Shi et al., 2015; Kanasaki et al., 2014). Additionally, WNT inhibition (via LGK-974) and mineralocorticoid receptor antagonism have shown impressive results in reducing DKD progression (Srivastava et al., 2021b). The conventional therapy of DKD involves reno-protective drugs, such as angiotensin-converting enzyme inhibitors (ACEi) and angiotensin receptor blockers (ARBs). The efficacy of ACEi and ARBs to the delay event or halt progession to kidney failure of proteinuric CKD is well established. An endogenous peptide, AcSDKP exerts significant antifibrotic effects by improving mitochondrial function, enhancing FAO, suppressing aberrant glycolysis-lactate pathway, inflammation, and partial EMT and EndMT processes in the diabetic kidney (Srivastava et al., 2020b; Nagai et al., 2014).

In this Research Topic, Li et al. demonstrated the antifibrotic effects of the phytochemical baicalin in a model of acute kidney injury (AKI). A study from Ke et al. showed that β-cryptoxanthin improved renal oxidative stress by activating Nrf2/HO-1 signaling pathway in diabetes. Muta et al. suggested that the addition of GLP-1 receptor agonists (GLP-1Ra) to patients already receiving SGLT2 inhibitors (SGLT2i) was associated with a slower annual decline in eGFR. A retrospective study by Otsuka et al. reported that transitioning from dulaglutide to tirzepatide improved glycemic control without elevating the risk of hypoglycemia in patients with type 2 diabetes undergoing hemodialysis. A study by Yang et al. suggested that SGLT2 inhibitors may be recommended as a therapeutic strategy to maximize renal and cardiovascular protection in DKD while posing a minimal risk of hyperkalemia than non-steroid mineralocorticoid receptor antagonists (ns-MRAs).

## Conclusion

Our first Research Topic provided foundational insights into the design of potential therapeutic agents for patients with DKD (Bhatia and Srivastava, 2023). This Research Topic offers a comprehensive overview of emerging pathophysiologic mechanisms and therapeutic targets that can be used for diverse phenotypes of DKD. We addressed key regulatory pathways involved in modulating the progression of kidney fibrosis and discussed their potential implications for future therapeutic strategies. Additionally, we highlighted novel cell-specific biological pathways and clinical datasets that could be further investigated in larger cohorts to improve DKD management. Our insights aim to bridge the gap between mechanistic understanding and the development of targeted therapies for DKD.
